# Latent classes of self-management behaviors and a network model of influencing factors in community-dwelling older patients with chronic diseases: a healthy aging perspective

**DOI:** 10.3389/fpubh.2026.1826369

**Published:** 2026-07-03

**Authors:** Haojun Huang, Zhanying Zhao, Tianli Xie, Xinru Hou, Keying Guo, Shanyu Wu, Jinji Wu

**Affiliations:** 1 School of Nursing, Yanbian University, Yanji, China; 2 Department of Nursing, Linyi Central Hospital, Linyi, Shandong, China

**Keywords:** healthy aging, network analysis, older patients with chronic diseases, potential profile analysis, self-management behavior

## Abstract

**Objectives:**

To identify potential classes of self-management behaviors and constructs a network model linking cognitive reserve, psychological capital, social health, and self-management behaviors, providing a basis for precise interventions.

**Methods:**

From January to June 2024, a convenience sampling method was employed to select 394 older patients with chronic diseases from two community health service centers in Yanji City. Latent Profile Analysis (LPA) was conducted to identify the latent classes of self-management behaviors, and network analysis along with stability testing was performed using R 4.4.2.

**Results:**

According to latent profile analysis, self-management behaviors can be classified into three classes: “inefficient self-management class” (14.7%), “uneven self-management class “(39.1%), and “efficient self-management class” (46.2%). After adjusting for 12 covariates, the efficient self-management class scored significantly higher than the uneven and inefficient self-management classes in terms of cognitive reserve [OR = 1.556, 95%CI: (1.319–1.836), *p* < 0.05], psychological capital [OR = 1.876, 95%CI: (1.286–2.736), *p* < 0.05], and social health [OR = 1.277, 95%CI: (1.164–1.440), *p* < 0.05]. Centrality indicators show that the three dimensions with the highest intensity and expected impact are self-efficacy, occupational activity, and integrity. Furthermore, self-management behaviors exhibited the greatest density and mediation in the network model, demonstrating a significant “bridge” effect.

**Conclusion:**

Self-management behavior exhibits three types of heterogeneity, with psychological capital, cognitive reserve, and social health collaboratively influencing. Psychological capital serves as an interactive hub between cognitive reserve and social health, with the self-efficacy dimension having the most significant impact on network structure.

## 1 Introduction

Population aging has become a significant challenge faced globally (1). This trend is universal and irreversible, with not only developed countries experiencing serious issues related to aging populations, but many developing countries also gradually entering an aging society (2). According to the latest data, the global population aged 65 and above reached 761 million in 2021 and is projected to grow to 1.6 billion by 2050 (3). As population aging continues worldwide, this demographic shift may have profound social implications and significantly increase the burden of chronic diseases (4, 5).

“Healthy aging” is an important strategy to address the challenges posed by population aging and chronic diseases, providing effective means to tackle the social issues arising from aging. Within the conceptual framework of healthy aging, the core of basic healthcare is “everyone takes responsibility for their own health” (6), with self-management being a key manifestation. Chronic diseases, as lifestyle-related diseases, have poor self-management behaviors as significant contributing factors to their occurrence. Research both domestically and internationally (7, 8) indicates that the self-management levels of older patients with chronic diseases in the community are generally moderate, suggesting that there is still room for improvement in the self-management behaviors of older chronic disease patients. Most older patients have a fragmented understanding of chronic disease management knowledge, lack systematic guidance, and have insufficient awareness of disease consequences, leading to a weak sense of self-management. Furthermore, self-management behaviors are closely related to psychological functioning, family functioning, and the sense of meaning in life. In 2011, Michie et al. (9) proposed the Capacity, Opportunity, Motivation-Behavior (COM-B) theoretical model. This model posits that individuals need to possess the capacity, opportunity, and motivation simultaneously in order to achieve a change in behavior. Within the COM-B framework, capacity, opportunity, and motivation interact to influence behavior. Currently, the COM-B theoretical model has been widely applied in various fields, including public health and health promotion, chronic disease management, geriatric syndromes and older care, nursing research and clinical nursing pathways, digital health and mobile healthcare, as well as environmental and food safety behaviors. The model is also frequently utilized in studies involving patients with chronic diseases such as hypertension, diabetes, chronic heart failure, chronic obstructive pulmonary disease, urinary incontinence, and populations at high risk for stroke (10). In the COM-B theory model, “capability” refers to an individual’s ability to engage in relevant activities, which corresponds to cognitive reserve in this study. Cognitive reserve may play a significant protective role in the brain function of older patients with chronic diseases. Research indicates that cognitive reserve can help maintain cognitive function by preserving the physiological robustness of brain functional networks (11). Furthermore, as an important factor in the “psychological-biological bridge,” cognitive reserve can enhance patients’ self-management decision-making abilities, thereby improving their self-management behaviors (12). “Motivation” refers to the brain activity processes that inspire and guide behavior, which corresponds to the psychological capital described in this study. Healthy psychological capital encompasses elements such as hope, resilience, optimism, and self-efficacy, which can motivate older patients with chronic diseases to actively cope with health challenges, maintain an optimistic attitude, and lead an active lifestyle (13). Older adults with a positive attitude and an active lifestyle typically exhibit better self-management behaviors. “Opportunities” refer to the environment, encompassing all external factors that ensure or promote individual behavior, specifically social health in this study. Social isolation is prevalent among older patients with chronic diseases and is associated with various negative health outcomes, including depression, reduced medication adherence, and increased mortality rates (14). As an important external resource, the social environment can alleviate life stressors for older adults and enhance their well-being (15), thereby promoting better self-management. Existing studies (16, 17) have indicated a certain correlation between cognitive reserve, psychological capital, and social health with self-management behaviors. However, to the best of our knowledge, no studies have specifically explored the relationship of these factors in older patients with chronic diseases. High levels of self-management behaviors significantly contribute to maintaining patients’ physical and mental health and improving their quality of life (18). Given the trend of increasing global aging and the rising burden of chronic diseases (5), it is of great practical significance to deeply investigate the influencing factors of self-management behaviors in older patients with chronic diseases to effectively improve their self-management and promote health.

However, previous studies often assess the level of self-management behaviors in patients through overall mean analysis, neglecting individual heterogeneity, which may lead to errors in the results. Latent Profile Analysis (LPA) (19) explains the associations between external continuous variables through latent categorical variables, achieving local independence among manifest variables. The application of LPA in aging research is particularly well-justified by the dedifferentiation hypothesis from lifespan theory, which posits that individuals become increasingly heterogeneous with age. Unlike younger populations who may exhibit relatively homogeneous developmental patterns, older adults accumulate diverse life experiences, health conditions, and psychosocial resources, resulting in more varied developmental trajectories in later life (20–22). Therefore, a person-centered approach such as LPA is especially appropriate for studying older populations, as it can identify latent subpopulations with distinct characteristics that traditional variable-centered methods might overlook. This methodological choice allows us to capture individual heterogeneity in self-management behaviors among community-dwelling older patients with chronic diseases (23). Epslamp et al. (24) points out that network analysis is an analytical method that explores the interactions and inherent structures between different components or related behaviors of observed variables through visualization, providing new perspectives and methods for exploring the relationships between various factors.

Therefore, from the perspective of healthy aging and against the backdrop of increasing global population aging and chronic disease burden, this study aims to investigate the latent classes of self-management behaviors and their influencing factors among community-dwelling older patients with chronic diseases, and to construct a network model linking cognitive reserve, psychological capital, social health, and self-management behaviors, thereby providing new evidence for improving self-management behaviors in this population. Specifically, this study addresses three research questions: (1) What distinct latent classes of self-management behaviors exist among community-dwelling older patients with chronic diseases? (2) How do cognitive reserve, psychological capital, and social health differ across these latent classes after adjusting for relevant covariates? (3) What are the network structures and key centrality dimensions (e.g., strength, expected influence, bridge effects) linking cognitive reserve, psychological capital, social health, and self-management behaviors?

## 2 Materials and methods

### 2.1 Study design and participants

Using convenience sampling, this study selected 394 older patients with chronic diseases who visited outpatient clinics at two community health service centers in Yanji City from January to June 2024. Inclusion criteria were: (1) age ≥60 years; (2) long-term residents in the community with established health records (local residency for ≥6 months within the year); (3) patients diagnosed with chronic diseases based on the International Classification of Diseases, 10th Revision (ICD-10) classification standards (25), in conjunction with the epidemiological characteristics of chronic diseases in the Yanji region, including: type 2 diabetes, hypertension, chronic ischemic heart disease, post-stroke sequelae, and chronic lower respiratory diseases; (4) clear consciousness, normal cognitive function, and voluntary participation. Exclusion criteria included: (1) history of psychiatric disorders; (2) presence of severe organic dysfunction; (3) participation in other similar research projects within the last 3 months.

### 2.2 Ethical approval

This study was approved by the Ethics Committee of Yanbian University Medical College (Approval No: 10326) and employed a questionnaire survey method to collect data. After receiving permission from relevant departments, nursing master’s students who had undergone professional training and were familiar with the research objectives conducted the questionnaire collection. The researchers provided detailed explanations of the purpose and content of the survey to the respondents and committed to ensuring the anonymity and confidentiality of their information. Respondents completed the questionnaire after signing the informed consent form, and the questionnaires were collected on-site by the researchers on the same day (the respondents and researchers conducted the survey in separate rooms to protect patient privacy). For respondents who were illiterate, the researchers read the questionnaire content question by question, and the respondents answered each question, with the researchers filling out the responses on their behalf. This program is carried out in accordance with the ethical approval and consent protocol described in this section. The questionnaire collection process was strictly controlled to ensure the authenticity and accuracy of the data. Each questionnaire was checked for completeness immediately after collection. Any questionnaire with missing responses was considered invalid and excluded. A total of 452 questionnaires were distributed, and 394 valid questionnaires were returned (effective response rate: 87.2%). Thus, all 394 questionnaires included in the final analysis were complete, with no missing data. All valid questionnaires were double-entered and cross-checked, with 20% randomly selected for verification. Given the low exclusion rate (12.8%), complete-case analysis is unlikely to introduce meaningful bias into parameter estimation.

### 2.3 General information questionnaire

A general information questionnaire (GIS) was designed, covering 16 items: age, gender, ethnicity, employment status, living situation, number of children, education level, pension insurance, marital status, monthly family income, medical insurance, number of hobbies, types of chronic diseases, duration of diagnosis, frequency of using the internet to search for self-management information on chronic diseases, and frequency of referencing online self-management information and knowledge to guide daily self-management behaviors.

### 2.4 Cognitive reserve index questionnaire

The questionnaire was developed by Nucci et al. (26) and encompasses three dimensions: education, occupational activity, and leisure activity, consisting of a total of 24 items. An example item from the leisure activity dimension is: “In your free time, how often do you read books or newspapers?” The total score is calculated using the standard calculation tool provided by Nucci. The Cognitive Reserve Index Questionnaire (CRIQ) has been widely applied across various populations, including the assessment of cognitive reserve in older patients with chronic diseases (27). Research indicates that higher CRIQ scores are associated with a lower risk of cognitive impairment, further confirming the potential application value of the CRIQ in assessing cognitive reserve (28). In this study, the Cronbach’s α coefficient of the scale was 0.727.

### 2.5 Psychological capital scale for older adults

This scale was developed by Shi (29) and includes four dimensions: self-efficacy, integrity and stability, resilience, and gratitude and contribution, comprising a total of 20 items. Example items include: “I have confidence in my abilities” (self-efficacy) and “Even when facing setbacks, I can bounce back” (resilience). The scale employs a 5-point Likert scoring method, with a maximum score of 100, indicating that higher scores reflect a higher level of psychological capital. This scale has been widely used among older patients with chronic diseases (30). In this study, its Cronbach’s α coefficient was found to be 0.961.

### 2.6 Social health scale for older adults

This scale was developed by Bao (31) and includes three dimensions: social support, social adaptation, and perceived environmental resources, comprising a total of 25 items with a maximum score of 125 points. An example item from the social support dimension is: “Is there someone you can rely on when you encounter difficulties?” A higher score indicates better social health status. The SHSE-l scale effectively assesses the social health status of older patients with chronic diseases, covering multiple dimensions such as social support, social adaptation, and perceived environmental resources, which aids in comprehensively understanding the social health level of patients (32). In this study, the Cronbach’s α coefficient of the scale was 0.904.

### 2.7 Chronic patient self-management behavior scale

The questionnaire was developed by Hanqiao (33) and consists of five dimensions: dietary habits, exercise management, medication compliance, emotion management, and family and social support, with each dimension containing four items. Example items include: *“Do you strictly follow the prescribed medication dosage and timing?” (medication compliance) and “How often do you engage in moderate-intensity exercise per week (e.g., brisk walking, Tai Chi)?*” (exercise management). A 5-point Likert scale was employed, with a total score of 100 points; a higher score indicates stronger self-management capabilities. The self-management survey for older patients with chronic diseases has been widely used among older chronic disease patients (34), and in this study, its Cronbach’s α coefficient was 0.951.

### 2.8 Statistical analysis

This study utilized SPSS 28.0 software for data analysis. For normally distributed continuous data, mean and standard deviation were used for description; categorical data were described using frequency and percentage. Inter-group comparisons were conducted using the Chi-square test (χ^2^ test) and analysis of variance. Additionally, multiple logistic regression analysis was employed to explore the associations between potential classes of self-management behaviors in older patients with chronic diseases in the community and cognitive reserve, psychological capital, and social health. The significance level was set at α = 0.05.

Latent Profile Analysis (LPA) was conducted using Mplus 8.7. The fit indices for the LPA model include (35): (1) Akaike Information Criterion (AIC) (36), Bayesian Information Criterion (BIC) (37), and Adjusted Bayesian Information Criterion (aBIC) (38); smaller values of these indices indicate better model fit; (2) Entropy (38) serves as an evaluation index for the accuracy of model classification, with values ranging from 0 to 1; the closer the Entropy is to 1, the more accurate the classification; (3) the Lo–Mendell–Rubin likelihood ratio test (LMR) (39) and the Bootstrapped Likelihood Ratio Test (BLRT) (38) indicate that when *p* < 0.05, the model with k classes is superior to the model with k-1 classes.

Following the latent profile analysis, multinomial logistic regression was conducted to examine associations between cognitive reserve, psychological capital, social health, and latent class membership. Specifically, each participant was assigned to the latent class corresponding to their maximum posterior probability, and this assignment served as the dependent variable. It should be noted that this approach does not account for the inherent classification uncertainty associated with latent class assignment. The three-class model in the present study achieved an Entropy of 0.959, indicating an exceptionally high level of classification certainty; thus, the impact of classification uncertainty on parameter estimation is minimal. While bias-corrected three-step approaches (e.g., BCH or R3STEP) can further adjust for classification error, the current approach is considered acceptable given the exploratory nature of the study and the excellent classification quality (40–42). The methodological implications are further discussed in the limitations section.

According to the network analysis operational guidelines by Epslamp et al. (24), this study utilizes the “bootnet” package in R version 4.4.2 to construct and evaluate a network model of cognitive reserve, psychological capital, social health, and self-management behaviors. Data visualization and centrality index estimation are implemented using the “qgraph” package. Network analysis visually represents the relationships between variables through nodes and edges, and assesses the intimacy of the network model as well as the centrality, independence, and mediating roles of each node by calculating indicators such as density, degree centrality, closeness centrality, and betweenness centrality (43). Based on previous studies (44), closeness and betweenness are considered unreliable when determining node importance. Therefore, this study uses strength and expected influence to evaluate the centrality of nodes, while closeness and betweenness are used to assess the bridging role of nodes. The bootnet version 1.5.6 and case-dropping bootstrap methods are employed to assess the stability of centrality indices, providing the related stability coefficient CS-C to evaluate the stability of each centrality indicator (24). The CS-C value should not be less than 0.25, and preferably greater than 0.50.

### 2.9 Sample size justification

A total of 394 community-dwelling older patients with chronic diseases were included in this study. The sample size was determined based on convenience sampling according to the number of eligible patients available at the community health service centers during the study period. Below, we provide a post-hoc justification for the adequacy of this sample size with respect to the analytical methods employed.

For latent profile analysis (LPA): Methodological studies have demonstrated that sample size requirements depend on the number of indicators, the expected number of classes, and class separation. Nylund et al. (38) showed via Monte Carlo simulations that *N* = 300 is sufficient for accurate identification of a three-class model under moderate class separation. Furthermore, Wurpts and Geiser (45) recommended a minimum of 50 cases per class to ensure stable parameter estimates. In our study, the smallest class (the inefficient self-management class) contained 58 cases (14.7%), meeting this criterion. Additionally, with five indicator dimensions, a rule of thumb of 10–20 cases per indicator would require 50–100 cases; our sample of 394 far exceeds this requirement.

For network analysis: The sample size requirement for network analysis depends on the number of nodes. Our network model included 11 nodes. Epskamp et al. (24) recommended that the stability coefficient (CS-C) of centrality indices should be at least 0.25 and preferably above 0.50. In our study, the CS-C value was 0.751, well above the recommended threshold, indicating high stability of the network estimates. Moreover, simulation studies by Fried et al. (46) suggested that when the ratio of sample size to number of nodes (N/p) exceeds 10, the estimation error of edge weights becomes acceptable. Our N/p ratio was approximately 35.8 (394/11) and 26.2 (394/25), far exceeding 10, further confirming the adequacy of the sample size.

In summary, although this study employed convenience sampling without *a priori* power analysis, the *post-hoc* justifications above confirm that the obtained sample is sufficient to support the main findings from both LPA and network analysis.

## 3 Results

### 3.1 Demographic and descriptive characteristics of the total sample

A total of 394 community-dwelling older patients with chronic diseases were included in this study. The mean age of the total sample was 70.07 ± 6.76 years, including 189 males (48.0%) and 205 females (52.0%). Regarding ethnicity, 295 participants (74.9%) were Han and 99 (25.1%) were ethnic minorities. In terms of living situation, 77 (19.5%) lived alone, 217 (55.1%) lived with a spouse, and 100 (25.4%) lived with descendants. Education levels were as follows: primary school or below, 149 (37.8%); middle school, 142 (36.0%); high school, 63 (16.0%); associate degree or above, 40 (10.2%). Married participants accounted for 304 (77.2%), while 90 (22.8%) were unmarried, widowed, or divorced. Monthly family income was <3,000 yuan in 126 (32.0%), 3,000–5,000 yuan in 89 (22.6%), and ≥5,000 yuan in 179 (45.4%). Medical insurance types included urban employee insurance (172, 43.7%), rural resident insurance (159, 40.4%), and self-paying (63, 16.0%). Regarding the number of chronic diseases, 206 (52.3%) had one, 118 (29.9%) had 2–3, and 70 (17.8%) had more than three. Disease duration was <3 years in 82 (20.8%), 3–6 years in 117 (29.7%), 6–10 years in 87 (22.1%), and >10 years in 108 (27.4%). The mean scores for the total sample were 83.73 ± 10.96 for cognitive reserve (CRIQ), 71.43 ± 17.26 for psychological capital (PCE), and 78.01 ± 12.07 for social health (SHSE). Additional details are presented in Table 1.

**Table 1:** Demographics and characteristics of participants (*N* = 394).

Variables	Total	Self-management behavior			*p*-value
		Inefficient (*n* = 58)	Uneven (*n* = 154)	Efficient (*n* = 182)	
Age, mean ± SD	70.07 ± 6.76	72.86 ± 7.26	70.66 ± 6.79	68.68 ± 6.22	<0.001
Age, *n* (%)					<0.001
60 ~ 70	187 (47.5)	15 (8.0)	70 (37.4)	102 (54.5)	
70 ~ 80	163 (41.4)	29 (17.8)	63 (38.7)	71 (43.6)	
≥80	44 (11.2)	14 (31.8)	21 (47.7)	9 (20.5)	
Sex, *n* (%)					<0.001
Male	189 (48.0)	45 (23.8)	71 (37.6)	73 (38.6)	
Female	205 (52.0)	13 (6.3)	83 (40.5)	109 (53.2)	
Ethnic group, *n* (%)					0.343
Han	295 (74.9)	49 (16.6)	113 (38.3)	133 (45.1)	
Ethnic minority	99 (25.1)	9 (9.1)	41 (41.4)	49 (49.5)	
Living situation, *n* (%)					0.005
Living alone	77 (19.5)	22 (28.6)	25 (32.5)	30 (39.0)	
Living with spouse	217 (55.1)	24 (11.1)	91 (41.9)	102 (47.0)	
Lives with descendants	100 (25.4)	12 (12.0)	38 (38.0)	50 (50.0)	
Education, *n* (%)					0.049
Primary school	149 (37.8)	31 (20.8)	58 (38.9)	60 (40.3)	
Middle school	142 (36.0)	17 (12.0)	55 (38.7)	70 (49.3)	
High school	63 (16.0)	6 (9.5)	30 (47.6)	27 (42.9)	
Associate degree	40 (10.2)	4 (10.0)	11 (27.5)	25 (62.5)	
Number of offspring, *n* (%)					0.116
1	90 (22.8)	16 (17.8)	34 (37.8)	40 (44.4)	
2	196 (49.7)	21 (10.7)	86 (43.9)	89 (45.4)	
≥3	108 (27.4)	21 (19.4)	34 (31.5)	53 (49.1)	
Retired, *n* (%)					0.188
Yes	295 (74.9)	49 (16.6)	113 (38.3)	133 (45.1)	
No	99 (25.1)	9 (9.1)	41 (41.4)	49 (49.5)	
Married status, *n* (%)					<0.001
Married	304 (77.2)	18 (5.9)	123 (40.5)	163 (53.6)	
Not married or living with a partner	90 (22.8)	40 (44.4)	31 (34.4)	19 (21.1)	
Income, *n* (%)					<0.001
<3,000	126 (32.0)	49 (38.9)	71 (56.3)	6 (4.8)	
3,000 ~ 5,000	89 (22.6)	3 (3.4)	68 (76.4)	18 (20.2)	
≥5,000	179 (45.4)	6 (3.4)	15 (8.4)	158 (88.3)	
Hobbies, *n* (%)					<0.001
0	71 (18.0)	18 (25.4)	5 (7.0)	48 (67.6)	
1 ~ 2	181 (45.9)	21 (11.6)	83 (45.9)	77 (42.5)	
3 ~ 4	142 (36.0)	19 (13.4)	66 (46.5)	57 (40.1)	
Medical Insurance, *n* (%)					<0.001
Urban employees	172 (43.7)	18 (10.5)	80 (46.5)	74 (43.0)	
Rural residents	159 (40.4)	23 (14.5)	71 (44.7)	65 (40.9)	
Self-paying	63 (16.0)	17 (27.0)	3 (4.8)	43 (68.3)	
Chronic disease, *n* (%)					<0.001
1	206 (52.3)	24 (11.7)	101 (49.0)	81 (39.3)	
2 ~ 3	118 (29.9)	17 (14.4)	41 (34.7)	60 (50.8)	
>3	70 (17.8)	17 (24.3)	12 (17.1)	41 (58.6)	
Duration of chronic disease diagnosis, *n* (%)					0.001
<3 years	82 (20.8)	8 (9.8)	32 (39.0)	42 (51.2)	
3 ~ 6 years	117 (29.7)	16 (13.7)	36 (30.8)	65 (55.6)	
6 ~ 10 years	87 (22.1)	16 (18.4)	27 (31.0)	44 (50.6)	
>10 years	108 (27.4)	18 (16.7)	59 (54.6)	31 (28.7)	
Consult self-management information, *n* (%)					<0.001
≤3/year	117 (29.7)	13 (11.1)	61 (52.1)	43 (36.8)	
≤3/month	167 (42.4)	23 (13.8)	70 (41.9)	74 (44.3)	
≥2/week	80 (20.3)	16 (20.0)	23 (28.7)	41 (51.2)	
≥1/day	30 (7.6)	6 (20.0)	0 (0.0)	24 (80.0)	
Refer to network information, *n* (%)					<0.001
Never	82 (20.8)	12 (14.6)	38 (46.3)	32 (39.0)	
Sometimes	158 (40.1)	25 (15.8)	58 (36.7)	75 (47.5)	
Often	93 (23.6)	14 (15.1)	34 (36.6)	45 (48.4)	
Always	61 (15.5)	7 (11.5)	24 (39.3)	30 (49.2)	
CRIQ scores	83.73 ± 10.96	73.67 ± 5.24	83.02 ± 8.39	87.53 ± 12.05	<0.001
PCE scores	71.43 ± 17.26	41.10 ± 13.22	69.54 ± 11.67	82.69 ± 7.15	<0.001
SHSE scores	78.01 ± 12.07	66.43 ± 9.98	76.84 ± 8.96	82.69 ± 12.28	<0.001

CRIQ, Cognitive Reserve Index Questionnaire; PCE, Psychological capital scale for older adults; SHSE, Social Health Scale for older adults.

### 3.2 Identification of potential classes of self-management behaviors

Using the five dimensions of the Self-Management Behavior Scale as explicit indicators, we fitted latent class models ranging from one to five classes. As the number of classes increased, the AIC, BIC, and aBIC values showed a gradual decline, with a notable inflection point observed in the decline trend of AIC, BIC, and aBIC between the two-class and three-class models. The three-class model was selected as the optimal solution based on the following fit indices: AIC = 9329.472, BIC = 9416.952, aBIC = 9347.146, Entropy = 0.959, with significant LMR (*p* < 0.001) and BLRT (*p* < 0.001). The four-classes model only displayed horizontal differences compared to the three-classes model, without identifying any specific classes. Considering both the simplicity and interpretability of the models, the three-classes model was ultimately selected as the optimal model. Detailed fitting indices for each model can be found in Table 2.

**Table 2:** Comparison of latent class models’ goodness of fit (*N* = 394).

Model	AIC	BIC	aBIC	Entropy	*P*_BLRT_	*P*_LMR_	Latent class probability
1C	11061.049	11100.813	11069.083	–	–	–	1
2C	9894.512	9958.134	9907.366	0.948	<0.001	<0.001	0.518/0.482
3C	9329.472	9416.952	9347.146	0.959	<0.001	<0.001	0.147/0.391/0.462
4C	9211.887	9323.225	9234.381	0.935	<0.001	<0.001	0.457/0.117/0.140/0.287
5C	9135.006	9270.202	9162.321	0.915	<0.001	0.259	0.140/0.140/0.147/0.414/0.160

AIC, Akaike information criterion; BIC, Bayesian information criterion; aBIC, Adjusted Bayesian information criterion; Log(L), log-likelihood; LMR, Lo–Mendell–Rubin; BLRT, Bootstrap likelihood ratio test. Fit index selection and interpretation followed Nylund et al. (38).

### 3.3 The naming and characteristics of potential classes of self-management behaviors

In this study, self-management behavior was measured by five dimensions. The specific meanings of each dimension are as follows: Dietary habits refer to regular meal patterns, balanced nutrition, and controlled intake of oil, salt, and sugar; exercise management refers to the frequency and duration of moderate-intensity aerobic activities (e.g., brisk walking, Tai Chi) per week; medication compliance refers to taking medication on time and at the prescribed dosage without self-adjustment or discontinuation; emotion management refers to the ability to recognize and regulate negative emotions (e.g., anxiety, depression); family and social support refers to tangible help and emotional support from family members, friends, or the community.

**Figure 1:**
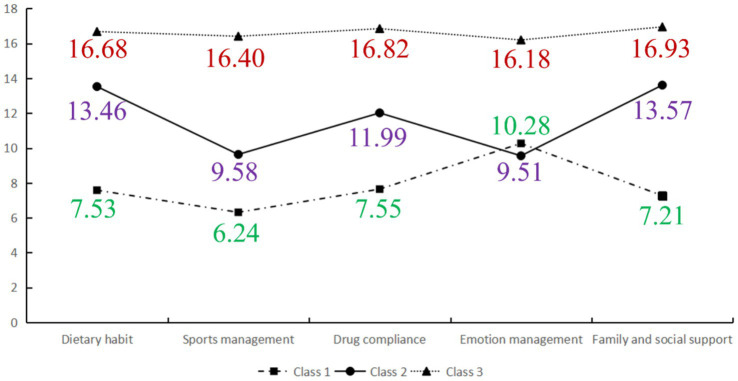
Depiction of three latent classes in terms of SMCS dimensions. The average values of the scores for each dimension of the inefficient self-management class are colored green, the average values of the scores for each dimension of the uneven self-management class are colored purple, and the average values of the scores for each dimension of the efficient self-management class are colored red.

Based on the latent profile analysis, the 394 community-dwelling older patients with chronic diseases were classified into three classes (see Figure 1).

Class 1: Inefficient self-management class (14.7%, *n* = 58). This class scored significantly lower than the other two classes on all five dimensions. Specifically, they had the poorest dietary quality (e.g., irregular meals, unbalanced nutrition), the lowest exercise frequency (almost no regular physical activity), the worst medication adherence (frequently missing doses or self-discontinuing medication), the weakest emotion management ability (difficulty coping with anxiety or depression), and the least social support (lack of tangible help from family and community). Overall, the self-management behaviors of this class were extremely poor.

Class 2: Uneven self-management class (39.1%, *n* = 154). This class showed a clear imbalance across dimensions. On dietary habits and family/social support, their scores approached those of the efficient class (e.g., being able to maintain relatively healthy eating and having some social support). However, on exercise management and emotion management, their scores were significantly lower, even comparable to those of the inefficient class. This indicates that while these patients performed relatively well in some aspects, they had notable deficiencies in exercise and emotion management.

Class 3: Efficient self-management class (46.2%, *n* = 182). This class scored the highest on all five dimensions. Specifically, they had good dietary habits (e.g., regular meals, controlled oil and salt intake), engaged in regular moderate-intensity exercise, strictly adhered to prescribed medications, were skilled at regulating negative emotions, and effectively utilized family and social support resources. Overall, this class demonstrated high-level, comprehensive self-management behaviors, representing an ideal health management pattern.

### 3.4 Analysis of influencing factors of potential classes of self-management behaviors

#### 3.4.1 Participants’ characteristics

Patients with chronic diseases in the efficient self-management class are typically younger (aged 60–70), predominantly female, and mostly belong to ethnic minorities. They usually live with their children, have an education level of college or above, are married, and have a monthly income of no less than 5,000 yuan. Furthermore, they often lack hobbies but possess self-paid health insurance, suffering from more than three types of chronic diseases, with a disease duration ranging from 3 to 6 years. These patients tend to seek help online and refer to health information (*p* < 0.05). As shown in Table 1, there are significant differences among the three classes of patients in terms of cognitive reserve scores, psychological capital scores, and social health scores (*p* < 0.05).

#### 3.4.2 The association between potential classes of self-management behaviors and cognitive reserves, psychological capital, and social health

Using the inefficient self-management class as a reference, multi-factor logistic regression analysis indicates that, compared to those in the uneven self-management class and the inefficient self-management class older patients with chronic diseases in the efficient self-management class have significantly higher scores in cognitive reserve [OR = 1.341, 95%CI: (1.244–1.446), *p* < 0.05], psychological capital [OR = 1.341, 95%CI: (1.273–1.412), *p* < 0.05], and social health [OR = 1.183, 95%CI: (1.134–1.233), *p* < 0.05], with the differences being statistically significant. After adjusting for 12 covariates, cognitive reserve, psychological capital, and social health remain positively correlated with the level of self-management behaviors. The cognitive reserve [OR = 1.556, 95%CI: (1.319–1.836), *p* < 0.05], psychological capital [OR = 1.876, 95%CI: (1.286–2.736), *p* = 0.001], and social health [OR = 1.277, 95%CI: (1.164–1.400), *p* < 0.05] levels of older patients with chronic diseases in the efficient self-management class are still significantly higher than those in the uneven self-management class and the inefficient class, with the differences being statistically significant (Table 3). Full regression outputs for Model 2, including all 12 covariates, are provided in Supplementary Tables S1–S3.

**Table 3:** Regression analyses with self-management as dependent variables (*N* = 394).

Variables	Uneven class (*n* = 154)			Efficient class (*n* = 182)		
	OR	95% CI	*P*	OR	95%CI	*P*
CRIQ scores						
Crude	1.284	1.193–1.383	<0.001	1.341	1.244–1.446	<0.001
Model 1	1.285	1.189–1.388	<0.001	1.345	1.243–1.456	<0.001
Model 2	1.429	1.221–1.672	<0.001	1.556	1.319–1.836	<0.001
PCE scores						
Crude	1.164	1.119–1.211	<0.001	1.341	1.273–1.412	<0.001
Model 1	1.183	1.128–1.240	<0.001	1.369	1.291–1.452	<0.001
Model 2	1.649	1.138–2.390	0.008	1.876	1.286–2.736	0.001
SHSE scores						
Crude	1.122	1.079–1.167	<0.001	1.183	1.134–1.233	<0.001
Model 1	1.119	1.074–1.166	<0.001	1.179	1.129–1.232	<0.001
Model 2	1.212	1.114–1.319	<0.001	1.277	1.164–1.400	<0.001

Crude: Unadjusted; Model 1: Adjusted by age, sex, ethnic; Model 2: Adjusted by age, sex, living situation, education, married status, income, hobbies, insurance, chronic disease, duration of chronic disease diagnosis, consult self-management information, refer to network information, CRIQ, Cognitive Reserve Index Questionnaire; PCE, Psychological capital scale for older adults; SHSE, Social Health Scale for older adults.

### 3.5 Network analysis of self-management behaviors and cognitive reserves, psychological capital, and social health

#### 3.5.1 The correlation between self-management behavior and various dimensions of cognitive reserve, psychological capital, and social health

Older patients with chronic diseases who exhibit a high level of self-management score significantly higher in dimensions such as education, occupational activity, leisure activity, self-efficacy, integrity and composure, resilience, gratitude and contribution, social support, social adaptation, and perceived environmental resources compared to those with uneven self-management and inefficient self-management. The differences are statistically significant (*p* < 0.05), as shown in Table 4.

**Table 4:** Comparison of the scores of various dimensions of cognitive reserves, psychological capital, and social health among different potential classes of self-management behaviors (*N* = 394).

Variables	Total	Self-management behavior			*P*-value
		Inefficient (*n* = 58)	Uneven (*n* = 154)	Efficient (*n* = 182)	
CRIQ scores					
Education (A1)	93.57 ± 10.97	88.38 ± 7.63	92.54 ± 10.27	96.09 ± 11.75	<0.001
Occupational activity (A2)	87.52 ± 8.93	80.05 ± 4.74	86.71 ± 6.33	90.58 ± 10.22	<0.001
Leisure activity (A3)	82.13 ± 10.83	72.00 ± 9.47	82.38 ± 7.59	85.14 ± 11.64	<0.001
PCE scores					
Self-efficacy (B1)	25.30 ± 6.01	14.55 ± 4.68	24.73 ± 3.87	29.21 ± 2.49	<0.001
Integrity and composure (B2)	17.71 ± 4.80	9.86 ± 3.57	17.17 ± 3.77	20.68 ± 2.29	<0.001
Resilience (B3)	17.83 ± 4.41	10.50 ± 2.99	17.43 ± 3.23	20.50 ± 2.47	<0.001
Gratitude and contribution(B4)	10.58 ± 3.05	6.19 ± 2.88	10.21 ± 2.48	12.30 ± 1.77	<0.001
SHSE scores					
Social support (C1)	41.53 ± 7.58	35.31 ± 5.88	41.12 ± 6.03	43.86 ± 8.08	<0.001
Social adaptation (C2)	16.23 ± 3.50	14.50 ± 3.46	15.77 ± 3.02	17.18 ± 3.61	<0.001
Perceived environmental resources(C3)	20.25 ± 4.59	16.62 ± 4.30	19.95 ± 4.44	21.65 ± 4.11	<0.001

CRIQ, Cognitive Reserve Index Questionnaire; PCE, Psychological capital scale for older adults; SHSE, Social Health Scale for older adults.

#### 3.5.2 The construction of a network model of self-management behavior and cognitive reserve, psychological capital and social health

**Figure 2:**
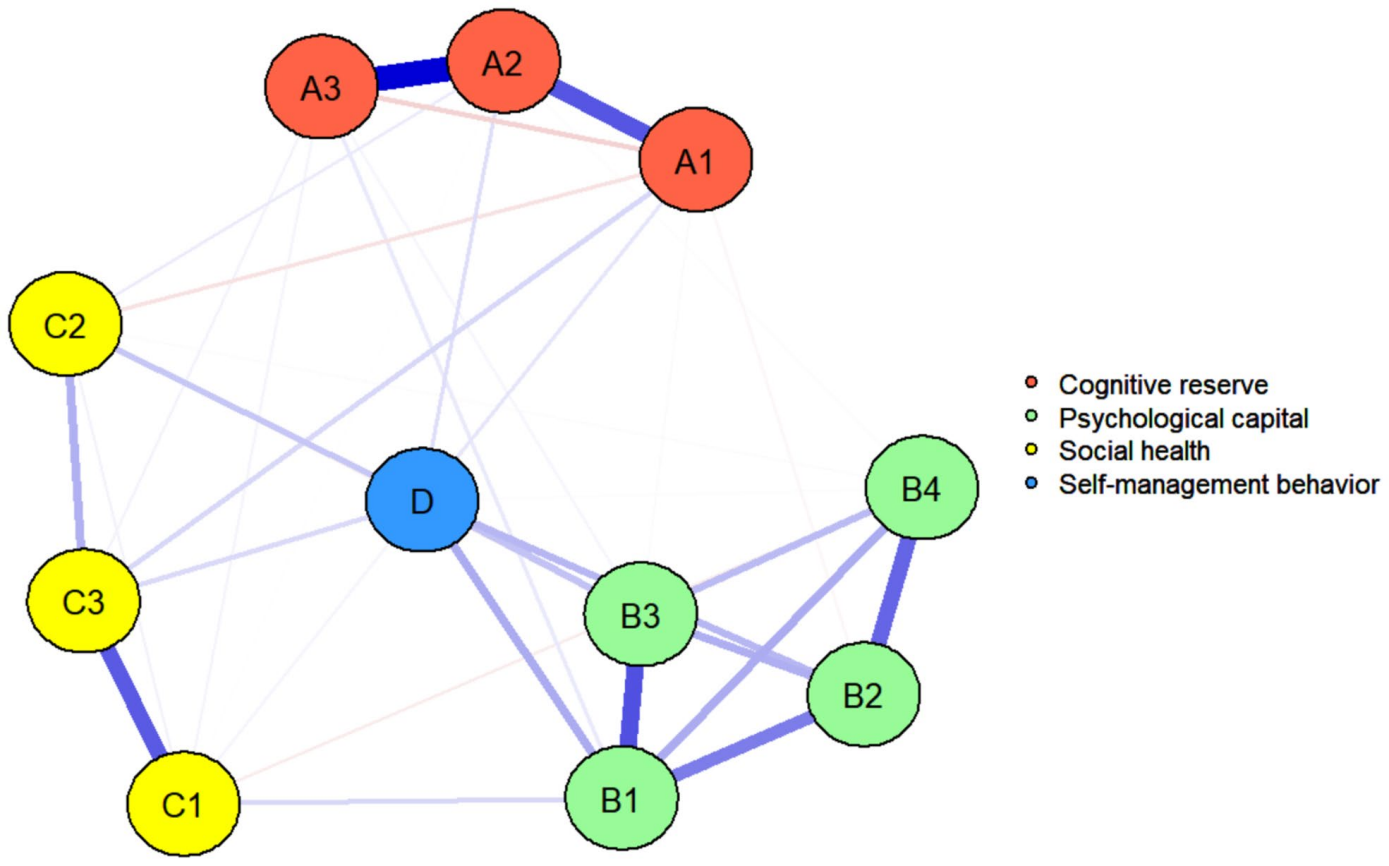
Network estimation of cognitive reserve, psychological capital, social health and self-management behavior.

This study constructs a network model that encompasses education, occupational activity, leisure activity, self-efficacy, integrity and composure, resilience, gratitude and contribution, social support, social adaptation, perceived environmental resources, and self-management behavior. In this model, cognitive reserve, psychological capital, and social health form three relatively concentrated but closely interconnected clusters, with mutual influences among the factors. The model includes a total of 11 nodes and 55 edges, of which 36 are non-zero edges (including 6 negative edges and 29 positive edges). Specifically, self-management behavior (D) is positively correlated with self-efficacy (B1), integrity and composure (B2), resilience (B3), and social adaptation (C2), with relatively tight connections (correlation coefficients of 0.20, 0.18, 0.15, and 0.14, respectively). Furthermore, within the dimensional network, there are significant positive correlations between self-efficacy (B1) and integrity and composure (B2), self-efficacy (B1) and resilience (B3), as well as between integrity and composure (B2) and resilience (B3) (correlation coefficients of 0.32, 0.43, and 0.20, respectively), as illustrated in Figure 2.

**Figure 3:**
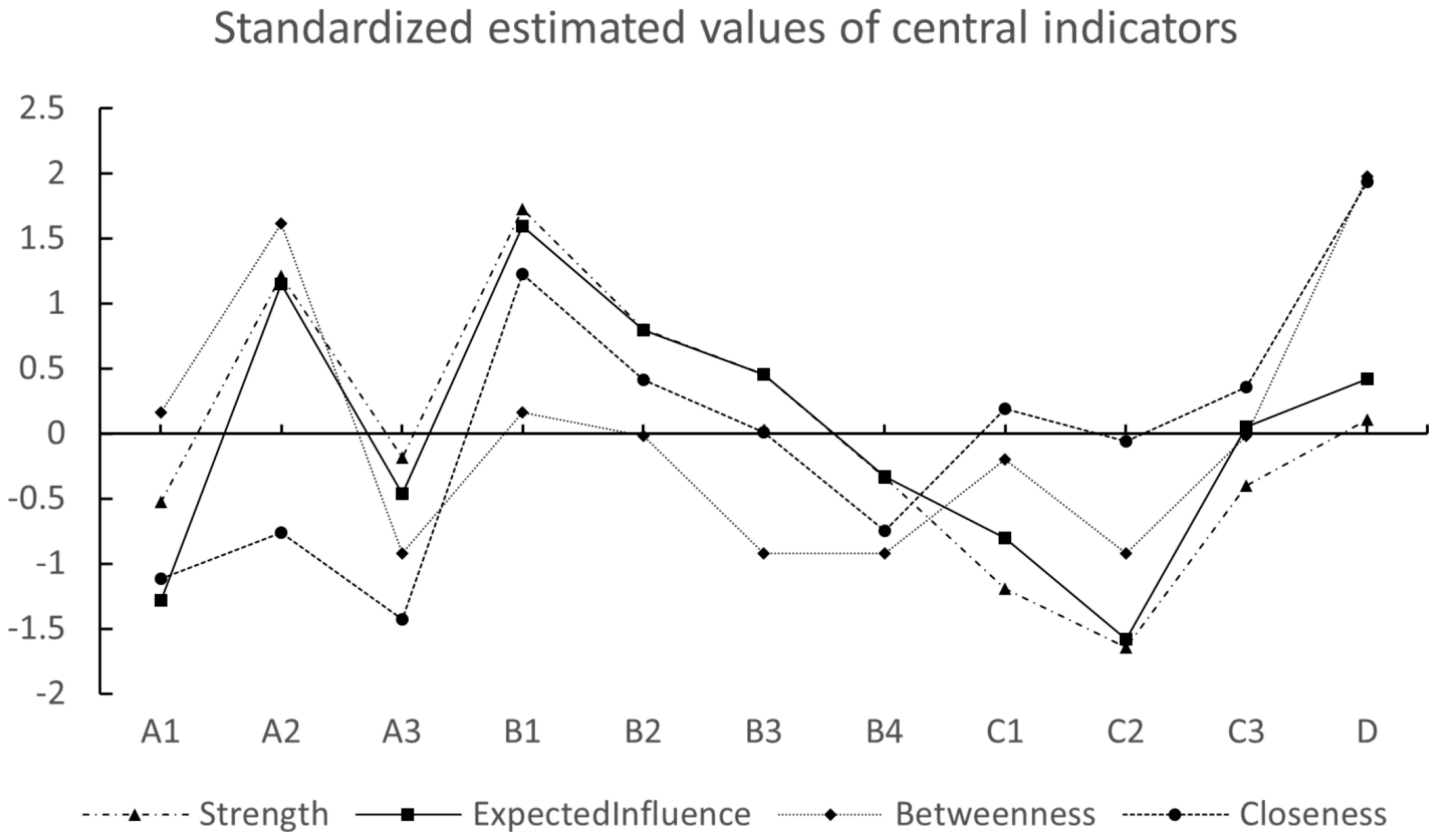
Central indicators of cognitive reserve, psychological capital, social health and self-management behavior network.

**Figure 4:**
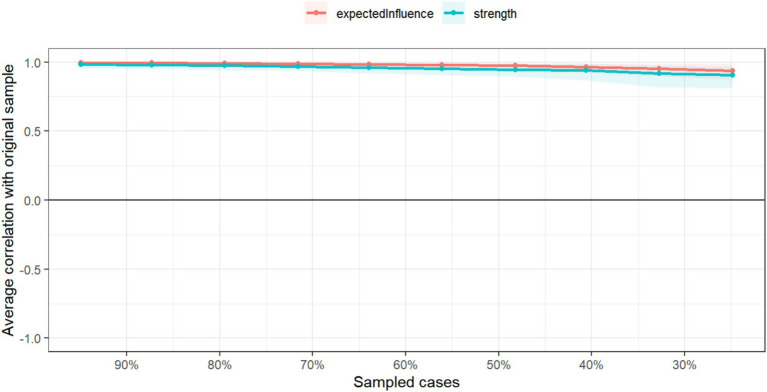
Analysis of the average correlation between centrality indicators and the original samples under different sampling ratios of cognitive reserve, psychological capital, social health and self-management behavior networks.

**Figure 5:**
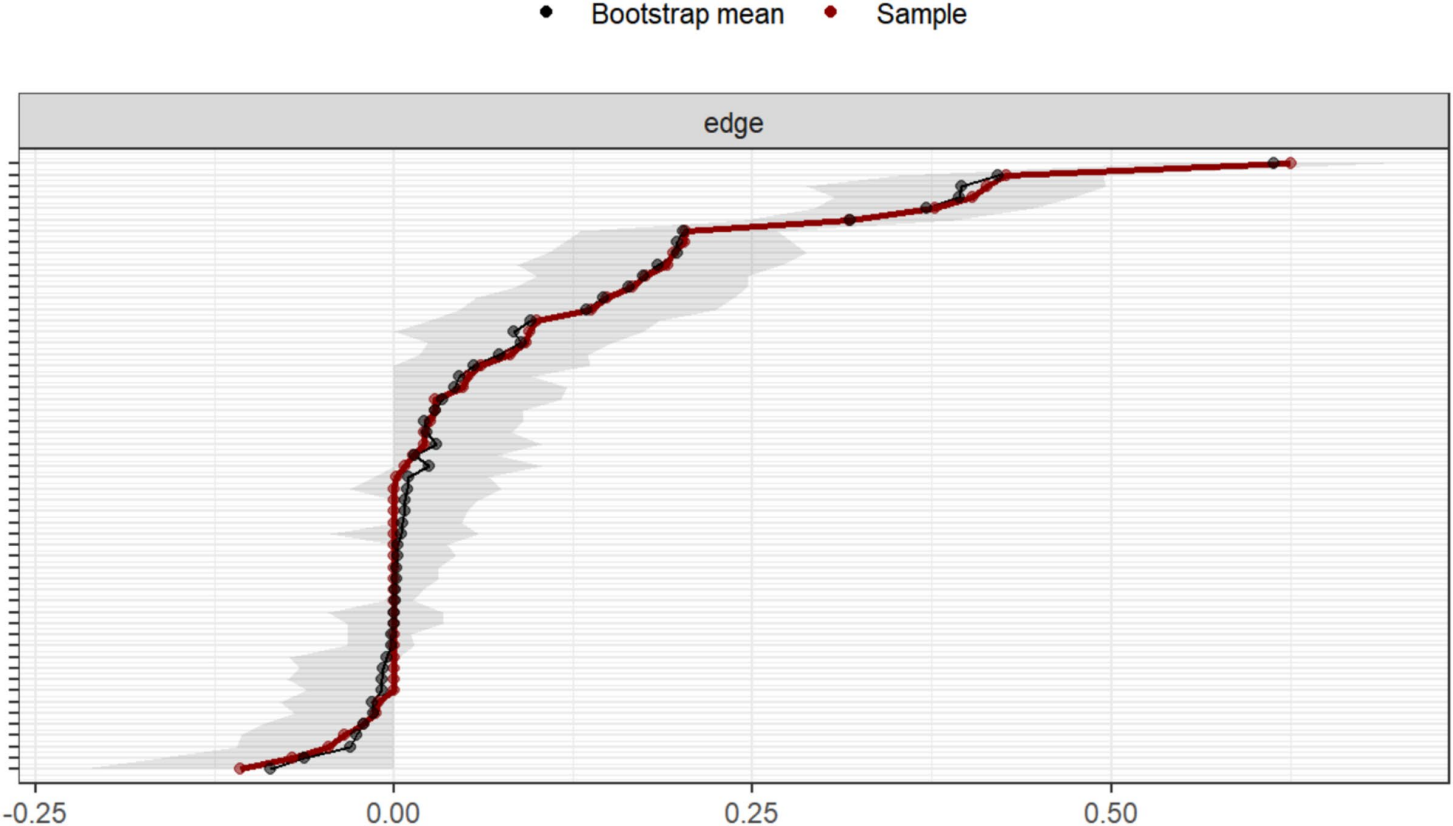
The Bootstrapped confidence interval of the edge weights of the network structure of the influencing factors of self-management behavior.

As shown in Figure 3, the top three dimensions ranked by the standardized estimates of strength and expected influence impact are self-efficacy (B1), occupational activity (A2), and integrity and composure (B2). This indicates that self-efficacy has the greatest impact on the network structure and is closely related to other dimensions. Self-management behavior (D) exhibits the highest closeness and betweenness standardized estimates, acting as a “bridge” within the entire network structure, closely connecting with occupational activity (A2) in cognitive reserve, self-efficacy (B1) in psychological capital, and social adaptation (C2) in social health. This suggests that these three influencing factors—cognitive reserve, psychological capital, and social health—jointly affect self-management behavior. The bootstrap results for the edge weight of the network and the stability assessment of centrality indicators reveal that the CS-C calculation results for intensity and expected impact are both 0.751, indicating good stability of the network structure (24), with high accuracy for intensity and expected impact. The stability assessment of centrality indicators shows that after extracting samples of different proportions, both intensity and expected impact remain stable, as seen in Figure 4. Furthermore, the 95% confidence interval for edge weights is narrow, indicating high precision in the estimation of edge weights (47), as shown in Figure 5.

#### 3.5.3 Sensitivity analysis: network model based on SMCS subscales

**Figure 6:**
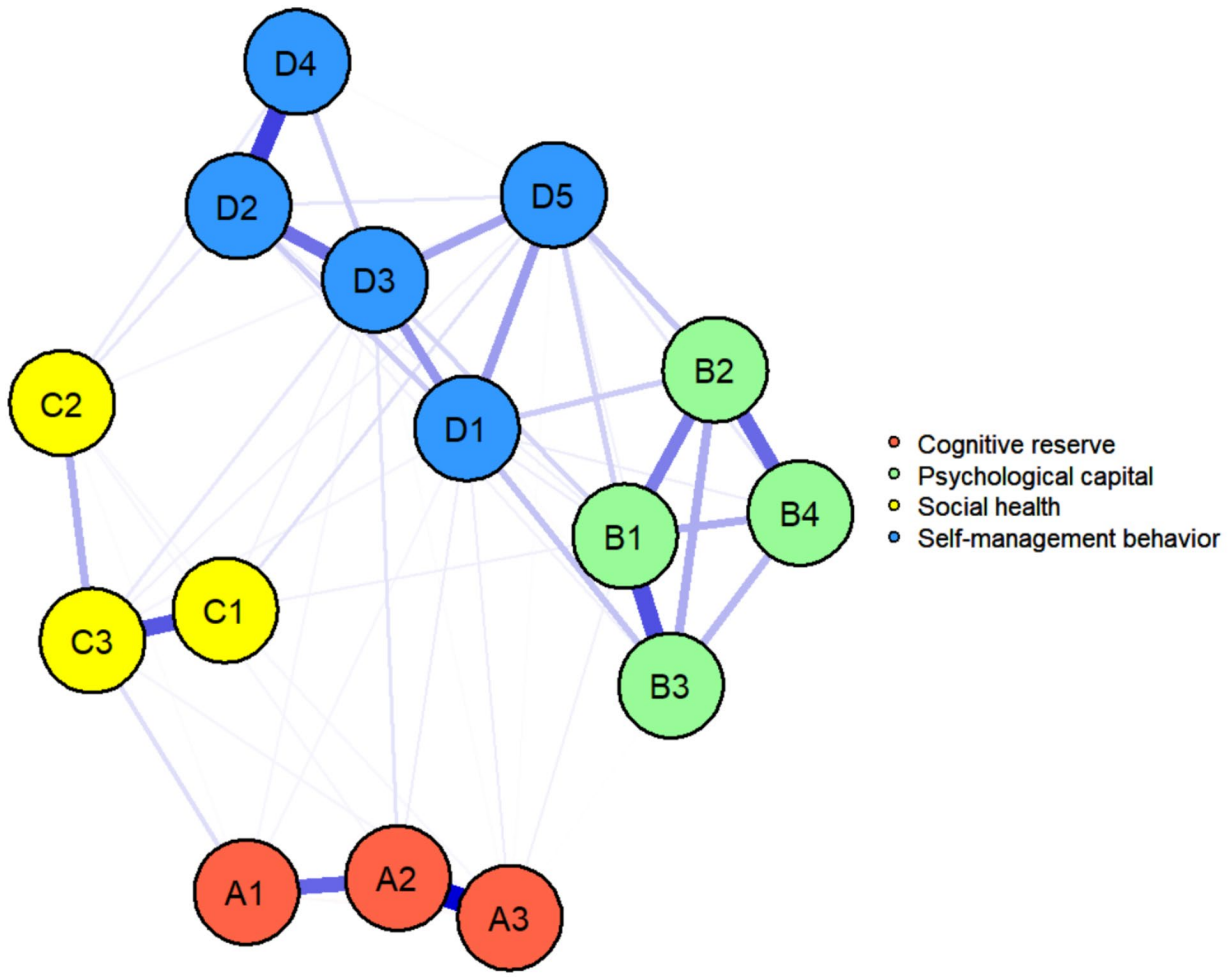
Network estimation of cognitive reserve, psychological capital, social health and specific dimension of self-management behaviors.

To examine the potential impact of granularity asymmetry on centrality estimation, a supplementary network analysis was conducted in this study. The five subdimensions of self-management behaviors (dietary habits, exercise management, medication compliance, emotion management, and family and social support) were treated as independent nodes, while the remaining nodes (A1–A3, B1–B4, C1–C3) were kept unchanged. A network model consisting of 15 nodes and 105 edges was constructed, of which 57 were non-zero edges (including 2 negative edges and 55 positive edges). Specifically, dietary habits (D1) showed positive and relatively strong correlations with integrity and composure (B2) and resilience (B3), as did family and social support (D5) with integrity and composure (B2) (*r* = 0.11, 0.13, and 0.13, respectively). Furthermore, in this dimensional network, significant positive correlations were observed between self-efficacy (B1) and integrity and composure (B2), between self-efficacy (B1) and resilience (B3), and between integrity and composure (B2) and resilience (B3) (*r* = 0.29, 0.40, and 0.19, respectively), as shown in Figure 6.

**Figure 7:**
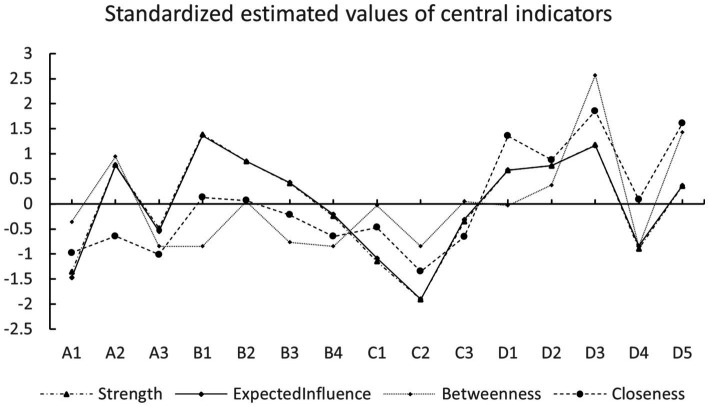
Network central indicators of cognitive reserve, psychological capital, social health and specific dimension of self-management behaviors.

**Figure 8:**
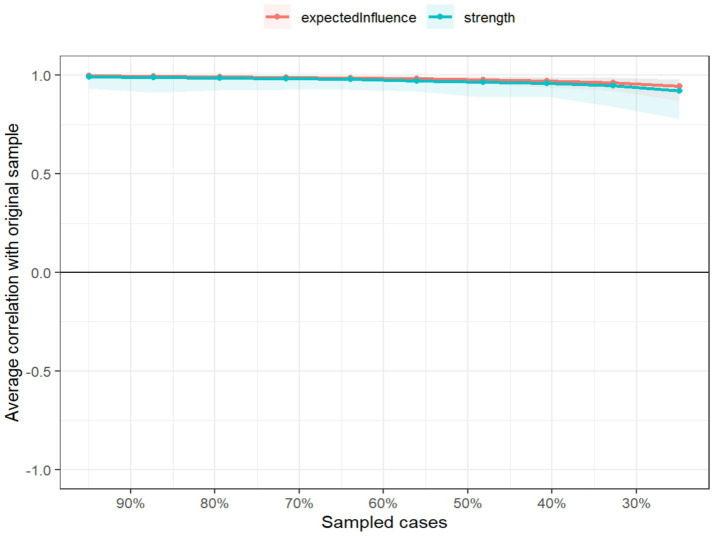
Analysis of the correlation between central indicators and the average values of the original sample under different sampling proportions of cognitive reserve, psychological capital, social health and the network of specific dimension of self-management behaviors.

**Figure 9:**
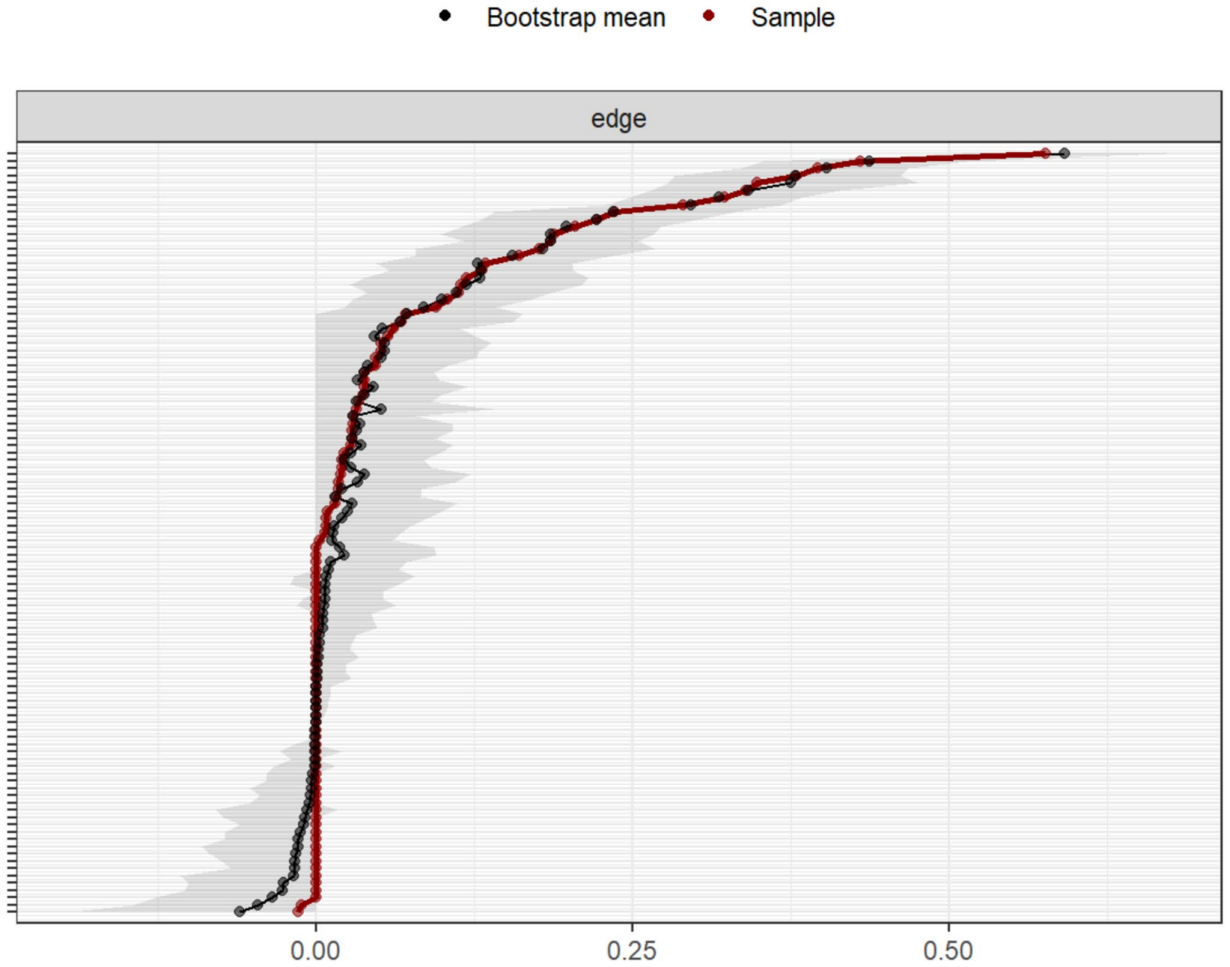
The self-calculated confidence intervals of the edge weights that affect the specific dimension of self-management behaviors within the network structure.

As shown in Figure 7, the three dimensions with the highest standardized estimates of strength and expected influence were self-efficacy (B1), medication compliance (D3), and integrity and composure (B2). This result indicates that self-efficacy exerts the greatest influence on the network structure and is closely associated with the other dimensions. Regarding bridging effects, the five subdimensions of self-management behaviors exhibited both closeness centrality and betweenness centrality above the network average. Notably, medication compliance (D3) and family and social support (D5) ranked first and second, respectively, in both closeness centrality and betweenness centrality. Through an evaluation of the bootstrap results for edge weights and the stability of centrality indices, the correlation stability (CS) coefficient for both strength and expected influence was found to be 0.751, indicating that the network structure has good stability and that the estimates of strength and expected influence are highly reliable (24). The stability assessment of centrality indices indicated that both strength and expected influence remained stable after subsampling with varying proportions, as shown in Figure 8. Furthermore, the 95% confidence intervals (95% CI) of the edge weights were narrow, indicating high precision in the estimation of edge weights (47), as presented in Figure 9.

In summary, the results of the supplementary sensitivity analysis indicate that each subdimension of self-management behaviors still exhibits a high level of bridging effects, and the main conclusions remain robust.

## 4 Discussion

This study employs latent profile analysis to identify the self-management behaviors of older patients with chronic diseases in the community into three classes: the inefficient self-management class (14.7%), the uneven self-management class (39.1%), and the efficient self-management efficiency class (46.2%). The findings indicate that the majority of older patients with chronic diseases in the community belong to the efficient self-management class, demonstrating a relatively high level of self-management. However, a subset of patients remains in the inefficient self-management class, warranting focused attention. For patients in the uneven self-management class, their self-management behaviors exhibit varying levels across different dimensions, necessitating particular attention to those dimensions with lower scores. Univariate analysis reveals that older patients in the efficient self-management class typically possess the following characteristics: relatively younger age, female gender, cohabitation with children, educational attainment of college degree or higher, marital status, monthly income of ≥5,000 yuan, lack of specific hobbies, private medical insurance, shorter duration since chronic disease diagnosis, and a higher number of chronic disease diagnoses. Our findings also indicate that the frequency of consulting online self-management information and the frequency of referencing online information significantly differed across the three self-management profiles (see Table 1). However, this finding requires cautious interpretation: the differences across subgroups should not be simplistically understood as “more information access leads to better self-management behavior.” Recent research highlights that psychological and social factors including individuals’ epistemic dispositions, critical appraisal capacity, and the ability to integrate health information into personal experience and daily practice significantly shape how individuals engage with, process, and use health information (48, 49). Specifically, epistemic trust: the subjective judgment of whether an information source is reliable and credible represents a key psychological process determining whether health information is effectively adopted. Nimbi et al. (48) demonstrated that lower levels of epistemic trust are significantly associated with skeptical attitudes toward health information and endorsement of false health beliefs. In older adults with chronic diseases, a population often disconnected from digital technology due to their generational background, inherent distrust toward online health information platforms is common. In our study, although patients in the efficient class used online information more frequently, the synergistic effects of self-efficacy and social support may have enhanced their epistemic trust, enabling more effective information filtering and application. Conversely, even when exposed to information, patients in the inefficient class may have dismissed it as unreliable or irrelevant due to insufficient epistemic trust. Second, critical appraisal capacity: the ability to discern the authority, accuracy, and relevance of health information sources, represents another key bottleneck limiting the rational use of health information. Research has shown that middle-aged and older adults with chronic diseases generally exhibit weak capacity to identify authoritative health information, making it difficult for them to make correct choices among numerous information channels (49). In our study, patients in the efficient class may have possessed stronger information discrimination abilities, tending to utilize professional healthcare channels or verified reliable sources, whereas those in the inefficient class likely lacked such appraisal capacity and were unable to distinguish credible information from misinformation despite having comparable exposure. In summary, the use of health information is a complex psychological process involving epistemic trust, critical appraisal, and practical integration, not merely a matter of frequency accumulation. Future self-management interventions for older adults with chronic diseases should focus not only on increasing the frequency of information access but also on cultivating epistemic trust and critical appraisal capacity to facilitate the translation of information into effective behavioral practices.

Notably, foreign research (50) indicates that as the number of chronic diseases increases, self-management abilities tend to decline. This suggests that the relationship between the number of chronic diseases and the level of self-management is not simply a positive or negative correlation. A study (51) points out that even with a higher number of chronic diseases, patients may still exhibit good self-management behaviors if they possess high health literacy and self-efficacy. The reason for this outcome in our study may be that older individuals with multiple chronic diseases often have higher health literacy and self-efficacy. Future research needs to further analyze the association between the two and consider the impact of additional confounding factors. Our study was conducted in Yanji, an area with a mixed Han and Korean-Chinese population. Although we did not find a significant association between ethnic group and self-management profiles (*p* = 0.343), this should not be interpreted as evidence that cultural and linguistic factors are irrelevant. Rather, it may reflect the homogenizing influence of standardized chronic disease management services provided by the community health centers, the universal application of evidence-based guidelines for the included chronic diseases, and the high Chinese language proficiency of Korean-Chinese participants in this sample.

In the multifactorial analysis, researchers found that after adjusting for 12 covariates, older patients with chronic diseases in the “efficient self-management class” significantly outperformed those in the “inefficient self-management class” and the “uneven self-management class” in terms of cognitive reserve, psychological capital, and social health levels. Network analysis results further revealed that within the dimensions of cognitive reserve, psychological capital, and social health, self-efficacy, integrity and composure, resilience, and social adaptation were positively correlated with self-management behaviors. Among these, the self-efficacy dimension within psychological capital exhibited the highest strength and expected influence impact in terms of centrality metrics, indicating the greatest influence on the network. Multiple studies (7, 52, 53) have shown that higher self-efficacy helps older patients with chronic diseases better manage their conditions, enhance quality of life, reduce disability and mortality rates, and improve physical, psychological, and social capabilities, thereby improving chronic disease prognosis and survival outcomes. Older individuals with high self-efficacy are more likely to engage in proactive self-management behaviors, such as appropriate medication adherence, healthy eating, and regular exercise, allowing them to maintain a positive mindset in the face of chronic disease challenges and reduce negative emotions such as anxiety and depression (54). An interventional study targeting older diabetic patients (55) demonstrated that empowerment programs effectively enhance self-efficacy and self-management capabilities among the older adults. Therefore, to improve self-efficacy among older patients with chronic diseases, enhance psychological capital, and boost self-management abilities, a variety of measures, such as health education, psychological interventions, and social support, can be implemented to improve chronic disease prognosis.

Integrity and stability in older patients with chronic diseases are more likely to foster confidence in disease management and enhance self-efficacy, thereby achieving better self-management (56). The health concepts and requirements within occupational activities significantly influence older patients with chronic diseases in terms of their health awareness and self-management behaviors (57). However, prolonged sedentary occupational activities increase the risk of chronic diseases (58). This finding may explain the results of the univariate analysis in this study: this population who exhibit high self-management behaviors often suffer from multiple chronic conditions, which may be related to their long-term engagement in high-risk occupational activities. Although some older patients with chronic diseases demonstrate high levels of self-management behaviors, they are still troubled by chronic illnesses. It is noteworthy that the course of some chronic diseases—particularly type 2 diabetes and early-stage hypertension—can be favorably modified, and in some cases clinical remission may be achieved, through improvements in lifestyle habits and self-management behaviors (59). However, the reversibility of chronic conditions is highly heterogeneous; for example, post-stroke sequelae and chronic ischemic heart disease are generally managed toward functional maintenance and secondary prevention rather than true reversal (60). Therefore, the potential for disease course modification through self-management should be interpreted with caution depending on the specific chronic condition and its stage.

The results of network analysis based on the COM-B theoretical model indicate that psychological capital serves as a core motivational component for self-management behaviors among these patients, playing a crucial driving role in enhancing their health behaviors. Furthermore, as a higher-order psychological factor, psychological capital interacts across dimensions with cognitive reserve and social health, being associated with behavior change via two pathways, though causal mediation cannot be inferred. This finding confirms that the mechanisms influencing self-management behaviors in community-dwelling older patients with chronic diseases align closely with the three-dimensional framework of capability-opportunity-motivation in the COM-B model, providing a theoretical basis for designing “motivation-empowerment” precision interventions. In this study, self-management behavior was initially treated as an aggregate node, whereas cognitive reserve, psychological capital, and social health were each disaggregated into subdimensional nodes. This asymmetry in node granularity may, to some extent, overestimate the bridging effects of the self-management node. Therefore, a supplementary sensitivity analysis was subsequently conducted, in which the five subdimensions of self-management behaviors (SMCS) were treated as independent nodes. The results showed that each subdimension still maintained moderate-to-high levels of bridging effects, indicating that the original main conclusions are robust.

Furthermore, the network models indicate that cognitive reserve, psychological capital, and social health are positively correlated with self-management behaviors. They are also closely interrelated with one another. This suggests that their relationship with self-management behaviors may not be a simple positive correlation, a phenomenon that requires further exploration in future research.

Our findings have several clinical implications for improving self-management behaviors in community-dwelling older patients with chronic diseases. First, the three identified classes (inefficient, uneven, and efficient) likely represent qualitatively different psychological organizations rather than mere differences in the quantity of cognitive or social resources. Therefore, intervention strategies must go beyond “one-size-fits-all” health education and move toward stratified, individualized approaches.

Specifically, we propose a three-level intervention framework:

Level 1: Behavioral-educational – applicable to all patients, including disease knowledge dissemination, medication adherence guidance, and standardized exercise prescriptions. However, for the inefficient self-management class, this level requires more structured support and ongoing supervision, as information provision alone rarely translates into behavioral change.

Level 2: Motivational-emotional – particularly targeted at the inefficient and uneven classes. The high centrality of self-efficacy and integrity/composure in our network analysis suggests that interventions should focus on: enhancing self-efficacy (e.g., via graded mastery experiences), helping patients regulate chronic illness-related negative emotions (e.g., fear of functional decline, stigma, depression/anxiety), and reframing the personal meaning of illness (from “uncontrollable” to “partially manageable”). Motivational interviewing and emotion-focused therapy are applicable at this level.

Level 3: Relational – especially for the inefficient class. These patients may exhibit avoidant or passive-dependent help-seeking patterns and lack trust in healthcare relationships. Interventionists should actively establish a stable, non-judgmental therapeutic alliance, encourage patients to express their fears and needs, and gradually train them to actively seek support. An effective patient-provider (or nurse–patient) relationship is itself a therapeutic ingredient.

It is important to emphasize that for patients in the inefficient self-management class, pure psychoeducation providing only knowledge and skills is unlikely to produce durable behavioral change. Their core difficulties likely lie in emotion dysregulation, negative illness narratives, and avoidant coping. These patients require intensified interventions that integrate psychotherapeutic elements, such as brief supportive psychotherapy or cognitive-behavioral techniques embedded in community nursing. Contemporary frameworks of personality functioning (e.g., PDM-3) provide theoretical tools to understand this psychological heterogeneity, highlighting the value of shifting from symptom-focused to person-centered formulations (61, 62).

In summary, from a healthy aging perspective, optimizing self-management behaviors in older patients with chronic diseases requires not only increasing cognitive reserve and social health resources but also deeply understanding their underlying psychological organization and providing multi-level interventions matched to their psychological profiles.

Several limitations of this study should be acknowledged. First, the cross-sectional design precludes any causal or directional inferences. Although our network analysis identified nodes with high centrality (e.g., self-efficacy), these should be interpreted as associations or potential intervention targets rather than as evidence of causal influence. Longitudinal or experimental studies are needed to test directional hypotheses. Second, we used convenience sampling from only two community health service centers in Yanji City. This sampling method may introduce selection bias and limits the generalizability of our findings to other regions, populations with different ethnic compositions, or healthcare systems with varying chronic disease management policies. Third, all data were collected via self-report questionnaires (CRIQ, PCE, SHSE, SMCS). Self-report measures are subject to recall bias, social desirability bias, and potential overestimation of self-management behaviors. Future studies should incorporate objective measures (e.g., actigraphy for physical activity, electronic monitoring for medication adherence) to complement self-reported data. Fourth, we did not perform an *a priori* sample size calculation. Although our final sample (*N* = 394) is comparable to or larger than many published latent profile and network analysis studies in similar populations, the absence of formal justification may affect statistical power. Of note, the network stability analysis yielded a CS-C coefficient of 0.751 for strength and expected influence, indicating acceptable stability. Nevertheless, future research should determine sample size based on planned complexity (e.g., number of latent classes, number of nodes in the network) and use appropriate power analysis tools when available. Despite these limitations, our study provides novel insights into the heterogeneity of self-management behaviors and their associations with cognitive reserve, psychological capital, and social health among community-dwelling older patients with chronic diseases.

## 5 Conclusion

Compared to previous studies, this research, based on the COM-B theoretical model, to our knowledge, the first to analyze the potential classes of self-management behaviors among older patients with chronic diseases in the community, identifying the heterogeneity of self-management behaviors among different patient classes. The present study employed a most likely class assignment approach (i.e., assigning each participant to the latent class corresponding to their maximum posterior probability) rather than a bias-corrected three-step approach when examining covariates associated with latent class membership. While classification uncertainty is a recognized methodological consideration in LPA research, studies have shown that when classification certainty is exceptionally high (Entropy = 0.959 in the present three-class model), the bias introduced by most likely class assignment is substantially reduced to an acceptable level (63). Empirical evidence has also indicated that the unadjusted modal assignment approach yields acceptable bias when class separation is large (41). Thus, this approach is deemed acceptable for the present exploratory analysis. Future studies using bias-corrected three-step methods may further eliminate any remaining classification measurement error. Additionally, the study constructs a network model of cognitive reserve, psychological capital, social health, and self-management behavior, visually demonstrating the strength of the associations between various dimensions and self-management behavior. This research provides a new theoretical basis for more effectively improving the self-management behaviors of these patients and optimizing chronic disease management strategies.

## Data Availability

The original contributions presented in the study are included in the article/Supplementary material, further inquiries can be directed to the corresponding authors.
