# Supplementary material for: Latent classes of self-management behaviors and a network model of influencing factors in community-dwelling older patients with chronic diseases: a healthy aging perspective
Source: Front Public Health. 2026 Jul 3;14:1826369. doi: 10.3389/fpubh.2026.1826369 (PMC13375910; doi:10.3389/fpubh.2026.1826369)
Supplement: Supplementary file 3 [file Table_1.DOCX]

| **Table S1** Complete regression output of Model 2 for cognitive reserve and self-management behaviors (N=394). | | | | | | |
| --- | --- | --- | --- | --- | --- | --- |
| Variables | Uneven class  (n=154) | | | Efficient class  (n=182) | | |
|  | OR | 95% CI | *P* | OR | 95%CI | *P* |
| CRIQ scores | 1.429 | 1.221-1.672 | <0.001 | 1.556 | 1.319-1.836 | <0.001 |
| Age | 0.929 | 0.836-1.033 | 0.173 | 0.908 | 0.804-1.024 | 0.116 |
| Sex, n (%) |  |  |  |  |  |  |
| Male | 0.122 | 0.028-0.523 | 0.005 | 0.105 | 0.022-0.512 | 0.005 |
| Female | - | - | - | - | - | - |
| Living situation, n (%) |  |  |  |  |  |  |
| Living alone | 0.796 | 0.105-6.066 | 0.826 | 2.954 | 0.296-29.460 | 0.356 |
| Living with spouse | 0.950 | 0.162-5.554 | 0.955 | 1.559 | 0.237-10.246 | 0.644 |
| Lives with descendants | - | - | - | - | - | - |
| Education, n (%) |  |  |  |  |  |  |
| Primary School | 1.135 | 0.082-15.809 | 0.925 | 0.896 | 0.043-18.554 | 0.943 |
| Middle School | 0.863 | 0.056-13.216 | 0.916 | 0.857 | 0.038-19.552 | 0.923 |
| High School | 0.583 | 0.018-18.790 | 0.761 | 0.329 | 0.007-14.807 | 0.567 |
| Associate Degree | - | - | - | - | - | - |
| Married status, n (%) |  |  |  |  |  |  |
| Not married or living with a partner | 0.233 | 0.051-1.072 | 0.061 | 0.117 | 0.018-0.749 | 0.023 |
| Married | - | - | - | - | - | - |
| Income, n (%) |  |  |  |  |  |  |
| < 3000 | 0.287 | 0.039-2.131 | 0.222 | <0.001 | 2.698E-5 | <0.001 |
| 3000~5000 | 15.004 | 1.008-223.371 | 0.049 | 0.082 | 0.005 | 0.077 |
| ≥5000 | - | - | - | - | - | - |
| Hobbies, n (%) |  |  |  |  |  |  |
| 0 | 0.017 | 0.002-0.147 | <0.001 | 0.185 | 0.021-1.630 | 0.128 |
| 1~2 | 0.218 | 0.044-1.072 | 0.061 | 0.066 | 0.010-0.438 | 0.005 |
| 3~4 | - | - | - | - | - | - |
| Medical Insurance, n (%) |  |  |  |  |  |  |
| Urban employees | 168.804 | 10.347-2753.965 | <0.001 | 1.428 | 0.133-15.381 | 0.769 |
| Rural residents | 293.793 | 16.540-2753.965 | <0.001 | 9.441 | 0.746-119.519 | 0.083 |
| Self-paying | - | - | - | - | - | - |
| Chronic disease, n (%) |  |  |  |  |  |  |
| 1 | 1.519 | 0.184-12.522 | 0.698 | 3.683 | 0.409-33.161 | 0.245 |
| 2~3 | 1.633 | 0.224-11.923 | 0.629 | 9.534 | 1.190-76.420 | 0.034 |
| >3 | - | - | - | - | - | - |
| Duration of chronic disease diagnosis, n (%) |  |  |  |  |  |  |
| <3 years | 3.052 | 0.449-20.760 | 0.254 | 8.639 | 0.980-76.170 | 0.052 |
| 3~6 years | 0.527 | 0.099-2.794 | 0.451 | 1.645 | 0.238-11.360 | 0.614 |
| 6~10 years | 0.645 | 0.099-4.186 | 0.646 | 1.519 | 0.168-13.733 | 0.710 |
| >10 years | - | - | - | - | - | - |

| **Table S1** Continued. | | | | | | |
| --- | --- | --- | --- | --- | --- | --- |
| Variables | Uneven class  (n=154) | | | Efficient class  (n=182) | | |
|  | OR | 95% CI | *P* | OR | 95%CI | *P* |
| Consult self-management information, n (%) |  |  |  |  |  |  |
| ≤3/year | 7351044329.666 | 692215342.016-78065089657.423 | <0.001 | 2.755 | 0.026-292.623 | 0.670 |
| ≤3/month | 1929311412.851 | 304282395.444-12232855345.852 | <0.001 | 0.521 | 0.007-38.539 | 0.766 |
| ≥2/week | 592050163.167 | 592050163.167-592050163.167 | <0.001 | 0.231 | 0.003-19.232 | 0.516 |
| ≥1/day | - | - | - | - | - | - |
| Refer to network information, n (%) |  |  |  |  |  |  |
| Never | 3.315 | 0.247-44.440 | 0.366 | 12.649 | 0.693-230.868 | 0.087 |
| Sometimes | 2.397 | 0.345-16.642 | 0.377 | 12.256 | 1.427-105.262 | 0.022 |
| Often | 1.239 | 0.134-11.454 | 0.850 | 5.700 | 0.512-63.463 | 0.157 |
| Always | - | - | - | - | - | - |
| Adjusted by age,sex,living situation,education,married status,income,hobbies,insurance,chronic disease,duration of chronic disease diagnosis,consult self-management information,refer to network information, CRIQ:Cognitive Reserve Index Questionnaire. | | | | | | |

| **Table S2** Complete regression output of Model 2 for psychological capital and self-management behaviors (N=394). | | | | | | |
| --- | --- | --- | --- | --- | --- | --- |
| Variables | Uneven class  (n=154) | | | Efficient class  (n=182) | | |
|  | OR | 95% CI | *P* | OR | 95%CI | *P* |
| PCE scores | 1.649 | 1.138-2.390 | 0.008 | 1.876 | 1.286-2.736 | 0.001 |
| Age | 0.809 | 0.572-1.145 | 0.232 | 0.807 | 0.565-1.151 | 0.237 |
| Sex, n (%) |  |  |  |  |  |  |
| Male | <0.001 | 4.999E－8-0.776 | 0.043 | 0.000 | 3.610E－8-0.608 | .038 |
| Female | - | - | - | - | - | - |
| Living situation, n (%) |  |  |  |  |  |  |
| Living alone | 0.415 | 0.002-75.714 | 0.741 | 1.186 | 0.005-276.126 | 0.951 |
| Living with spouse | 0.621 | 0.011-34.164 | 0.816 | 1.949 | 0.030-128.672 | 0.755 |
| Lives with descendants | - | - | - | - | - | - |
| Education, n (%) |  |  |  |  |  |  |
| Primary School | 70.183 | 0.113-43621.050 | 0.195 | 32.537 | 0.051-20910.489 | 0.291 |
| Middle School | 308.572 | 0.869-109633.080 | 0.056 | 302.084 | 0.967-94348.279 | 0.051 |
| High School | 5346.589 | 2.054-13919401.256 | 0.032 | 8746.791 | 3.467-22069267.128 | 0.023 |
| Associate Degree | - | - | - | - | - | - |
| Married status, n (%) |  |  |  |  |  |  |
| Not married or living with a partner | 2.789 | 0.011-677.998 | 0.714 | 1.469 | 0.005-437.001 | 0.895 |
| Married | - | - | - | - | - | - |
| Income, n (%) |  |  |  |  |  |  |
| < 3000 | 0.099 | 0.000-74.283 | 0.494 | 7.473E－5 | 8.051E－8-0.069 | 0.006 |
| 3000~5000 | 72.293 | 0.186-28080.237 | 0.159 | 0.606 | 0.002-237.283 | 0.870 |
| ≥5000 | - | - | - | - | - | - |
| Hobbies, n (%) |  |  |  |  |  |  |
| 0 | 1.215E-5 | 7.876E－10-0.187 | 0.021 | 0.000 | 1.181E－8-2.535 | 0.077 |
| 1~2 | 0.009 | 1.258E－5-5.762 | 0.152 | 0.003 | 4.166E－6-2.903 | 0.099 |
| 3~4 | - | - | - | - | - | - |
| Medical Insurance, n (%) |  |  |  |  |  |  |
| Urban employees | 33.717 | 0.123-9206.625 | 0.219 | 0.731 | 0.003-174.258 | 0.911 |
| Rural residents | 1265.812 | 0.244-6573625.974 | 0.102 | 135.044 | 0.029-630627.048 | 0.255 |
| Self-paying | - | - | - | - | - | - |

| **Table S2** Continued. | | | | | | |
| --- | --- | --- | --- | --- | --- | --- |
| Variables | Uneven class  (n=154) | | | Efficient class  (n=182) | | |
|  | OR | 95% CI | *P* | OR | 95% CI | *P* |
| Chronic disease, n (%) |  |  |  |  |  |  |
| 1 | 8.825 | 0.038-2027.215 | 0.432 | 11.116 | 0.045-2759.264 | 0.392 |
| 2~3 | 2.105 | 0.027-164.941 | 0.738 | 6.311 | 0.072-553.166 | 0.420 |
| >3 | - | - | - | - | - | - |
| Duration of chronic disease diagnosis, n (%) |  |  |  |  |  |  |
| <3 years | 1.178 | 0.005-298.128 | 0.954 | 3.227 | 0.011-949.154 | 0.686 |
| 3~6 years | 0.359 | 0.002-81.634 | 0.711 | 2.157 | 0.008-550.380 | 0.786 |
| 6~10 years | 0.008 | 7.478E－6-8.514 | 0.174 | 0.011 | 9.320E－6-13.569 | 0.215 |
| >10 years | - | - | - | - | - | - |
| Consult self-management information, n (%) |  |  |  |  |  |  |
| ≤3/year | 2680207000495.767 | 370904127.574-19367564368950404.000 | <0.001 | 2674.434 | 0.091-78239379.270 | 0.133 |
| ≤3/month | 115690774216.197 | 250330492.235-53466739585897.250 | <0.001 | 63.030 | 0.039-102979.181 | 0.272 |
| ≥2/week | 103805202.674 | 103805202.674-103805202.674 | <0.001 | 0.040 | 5.941E－5-26.539 | 0.331 |
| ≥1/day | - | - | - | - | - | - |
| Refer to network information, n (%) |  |  |  |  |  |  |
| Never | 5.895E－5 | 1.436E－10-24.208 | 0.140 | 9.177E－5 | 1.977E－10-42.612 | 0.163 |
| Sometimes | 0.001 | 4.971E－8-9.831 | 0.136 | 0.002 | 1.336E－7-30.815 | 0.207 |
| Often | 0.056 | 0.000-12.074 | 0.293 | 0.173 | 0.001-54.049 | 0.549 |
| Always | - | - | - | - | - | - |
| Adjusted by age,sex,living situation,education,married status,income,hobbies,insurance,chronic disease,duration of chronic disease diagnosis,consult self-management information,refer to network information, PCE: Psychological capital scale for older adults. | | | | | | |

| **Table S3** Complete regression output of Model 2 for social health and self-management behaviors (N=394). | | | | | | |
| --- | --- | --- | --- | --- | --- | --- |
| Variables | Uneven class  (n=154) | | | Efficient class  (n=182) | | |
|  | OR | 95% CI | *P* | OR | 95%CI | *P* |
| SHSE scores | 1.212 | 1.114-1.319 | <0.001 | 1.277 | 1.164-1.400 | <0.001 |
| Age | 0.938 | 0.861-1.021 | 0.140 | 0.921 | 0.832-1.021 | 0.117 |
| Sex, n (%) |  |  |  |  |  |  |
| Male | 0.063 | 0.014-0.286 | <0.001 | 0.041 | 0.008-0.211 | <0.001 |
| Female | - | - | - | - | - | - |
| Living situation, n (%) |  |  |  |  |  |  |
| Living alone | 0.560 | 0.100-3.139 | 0.510 | 1.584 | 0.215-11.647 | 0.652 |
| Living with spouse | 0.619 | 0.116-3.306 | 0.575 | 0.972 | 0.164-5.758 | 0.975 |
| Lives with descendants | - | - | - | - | - | - |
| Education, n (%) |  |  |  |  |  |  |
| Primary School | 0.423 | 0.040-4.479 | 0.475 | 0.199 | 0.012-3.414 | 0.265 |
| Middle School | 0.862 | 0.078-9.580 | 0.904 | 0.533 | 0.031-9.217 | 0.665 |
| High School | 0.509 | 0.041-6.399 | 0.601 | 0.235 | 0.013-4.321 | 0.329 |
| Associate Degree | - | - | - | - | - | - |
| Married status, n (%) |  |  |  |  |  |  |
| Not married or living with a partner | 0.088 | 0.020-0.381 | 0.001 | 0.049 | 0.009-0.276 | 0.001 |
| Married | - | - | - | - | - | - |
| Income, n (%) |  |  |  |  |  |  |
| < 3000 | 0.127 | 0.012-1.282 | 0.080 | <0.001 | 1.990E－5-0.003 | <0.001 |
| 3000~5000 | 3.137 | 0.253-38.845 | 0.373 | 0.027 | 0.002-0.311 | 0.004 |
| ≥5000 | - | - | - | - | - | - |
| Hobbies, n (%) |  |  |  |  |  |  |
| 0 | 0.021 | 0.003-0.159 | <0.001 | 0.220 | 0.031-1.583 | 0.133 |
| 1~2 | 0.631 | 0.151-2.642 | 0.529 | 0.259 | 0.046-1.462 | 0.126 |
| 3~4 | - | - | - | - | - | - |
| Medical Insurance, n (%) |  |  |  |  |  |  |
| Urban employees | 187.283 | 16.303-2151.452 | <0.001 | 2.436 | 0.280-21.220 | 0.420 |
| Rural residents | 705.618 | 46.932-10608.817 | <0.001 | 30.146 | 2.587-351.324 | 0.007 |
| Self-paying | - | - | - | - | - | - |
| Chronic disease, n (%) |  |  |  |  |  |  |
| 1 | 0.636 | 0.116-3.498 | 0.603 | 0.687 | 0.113-4.188 | 0.684 |
| 2~3 | 0.489 | 0.071-3.347 | 0.466 | 1.209 | 0.166-8.791 | 0.851 |
| >3 | - | - | - | - | - | - |

| **Table S3** Continued. | | | | | | |
| --- | --- | --- | --- | --- | --- | --- |
| Variables | Uneven class  (n=154) | | | Efficient class  (n=182) | | |
|  | OR | 95% CI | *P* | OR | 95% CI | *P* |
| Duration of chronic disease diagnosis, n (%) |  |  |  |  |  |  |
| <3 years | 4.591 | 0.755-27.938 | 0.098 | 15.327 | 1.814-129.506 | 0.012 |
| 3~6 years | 0.933 | 0.210-4.138 | 0.927 | 4.072 | 0.686-24.188 | 0.122 |
| 6~10 years | 1.236 | 0.240-6.369 | 0.800 | 4.222 | 0.589-30.268 | 0.152 |
| >10 years | - | - | - | - | - | - |
| Consult self-management information, n (%) |  |  |  |  |  |  |
| ≤3/year | 3255563695.191 | 388921712.163-27251486975.394 | <0.001 | 1.901 | 0.056-65.087 | 0.722 |
| ≤3/month | 2476722607.870 | 299614501.342-20473491265.802 | <0.001 | 1.032 | 0.037-28.688 | 0.985 |
| ≥2/week | 90178520.266 | 90178520.266-90178520.266 | <0.001 | 0.045 | 0.001-1.971 | 0.108 |
| ≥1/day | - | - | - | - | - | - |
| Refer to network information, n (%) |  |  |  |  |  |  |
| Never | 4.660 | 0.387-56.155 | 0.226 | 15.757 | 1.057-234.855 | 0.045 |
| Sometimes | 2.797 | 0.300-26.077 | 0.367 | 13.909 | 1.352-143.070 | 0.027 |
| Often | 4.110 | 0.397-42.563 | 0.236 | 32.460 | 2.567-410.449 | 0.007 |
| Always | - | - | - | - | - | - |
| Adjusted by age,sex,living situation,education,married status,income,hobbies,insurance,chronic disease,duration of chronic disease diagnosis,consult self-management information,refer to network information, SHSE: Social Health Scale for the Elderly. | | | | | | |
